# Neighbourhood Environmental Attributes Associated with Walking in South Australian Adults: Differences between Urban and Rural Areas

**DOI:** 10.3390/ijerph14090965

**Published:** 2017-08-26

**Authors:** Narelle M. Berry, Neil T. Coffee, Rebecca Nolan, James Dollman, Takemi Sugiyama

**Affiliations:** 1 Norwich Medical School, University of East Anglia, Norwich NR4 7UQ, UK; 2 Centre for Research and Action in Public Health (CeRAPH), UC Health Research Institute, University of Canberra, Canberra 2601, Australian Capital Territory, Australia; neil.coffee@canberra.edu.au; 3 Prevention and Population Health Branch, South Australian Department for Health and Ageing, Adelaide 5001, South Australia, Australia; rebecca.nolan@sa.gov.au; 4 School of Health Sciences, University of South Australia, Adelaide 5001, South Australia, Australia; James.dollman@unisa.edu.au; 5 Institute for Health and Ageing, Australian Catholic University, Melbourne 3000, Victoria, Australia; takemi.sugiyama@acu.edu.au

**Keywords:** walking, walkability, rural, urban, environment

## Abstract

Although the health benefits of walking are well established, participation is lower in rural areas compared to urban areas. Most studies on walkability and walking have been conducted in urban areas, thus little is known about the relevance of walkability to rural areas. A computer-assisted telephone survey of 2402 adults (aged ≥18 years) was conducted to determine walking behaviour and perceptions of neighbourhood walkability. Data were stratified by urban (n = 1738) and rural (n = 664). A greater proportion of respondents reported no walking in rural (25.8%) compared to urban areas (18.5%). Compared to urban areas, rural areas had lower walkability scores and urban residents reported higher frequency of walking. The association of perceived walkability with walking was significant only in urban areas. These results suggest that environmental factors associated with walking in urban areas may not be relevant in rural areas. Appropriate walkability measures specific to rural areas should be further researched.

## 1. Introduction

The health benefits of walking have been well established [1,2,3,4,5]. Walking is the most popular form of physical activity, and is often a key element in public health strategies to promote population health [6]. However, it has recently been demonstrated that walking participation differs between adults living in urban and rural areas, with those in rural areas walking less [7,8]. In Australia, 39% of people in outer regional and remote areas were identified as sufficiently active for health, compared to 45% in major cities [9]. Lower prevalence of physical activity in rural areas is one of the risk factors that may contribute to poorer health outcomes in rural areas compared to urban areas [10].

The built environment can play a pivotal role in residents’ walking behaviours, and living in walkable neighbourhoods is known to be associated with more frequent participation in walking for transport [11], longer walking duration [12], and more daily steps [13]. A walkable environment is conceptualised as having more destinations (such as shops, services and recreational resources) close by; and easy-to-walk, well-connected streets, to get to such local destinations [14].

It is possible, however, that environmental influences on walking differ between urban and rural areas due to differences in the way residents move within their environments. A review on physical activity in rural settings reported aesthetics, access to recreational facilities (parks, trails), and safety from crime as relevant environmental factors [15]. These attributes are known to be associated with recreational physical activity [14,16]. Supporting this notion, Kegler et al., (2014) suggested that most walking in rural areas is for leisure rather than for transport to local destinations [17]. Given the associations found between perceptions of walkability elements and recreational walking in a large-scale international study [18], it is possible that perceived walkability may be associated with walking in rural areas. While there are studies examining environmental predictors of activity in rural areas, there has been little research that compares how measures of walkability are related to walking between urban and rural areas [15,19,20].

Therefore, this study aimed to examine if there were differences in perceptions of walkability between individuals living in urban and rural areas in South Australia, and how urban and rural areas differed in associations between perceived walkability and walking behaviour. Because of the known differences in walking behaviour between men and women [21], the analysis was also stratified by sex.

## 2. Materials and Methods

### 2.1. Data Source

Data were collected as part of the 2013 South Australian Physical Activity Survey (SAPAS). The SAPAS is a Computer Assisted Telephone Interview survey managed by Population Research and Outcome Studies at the University of Adelaide, for the South Australian Department for Health and Ageing.

The survey content and methodology were approved by the South Australian Department for Health and Ageing Human Research Ethics Committee (Protocol number: HREC/14/SAH/62), and participants gave informed consent before participating in the survey. Trained interviewers conducted the SAPAS between September and November 2013. The questionnaire was pilot tested (n = 46) prior to the commencement of the survey.

All households in South Australia with a listed landline telephone number were eligible for selection in the sample. Telephone numbers were randomly selected from the Integrated Public Number Database (IPND). Within each household, the person who had the most recent birthday, and was 18 years or older, was selected for interview. There was no replacement for non-contactable persons.

The overall response rate in the SAPAS was 53.1%. In total, 4522 contacts were made, of whom 2402 were invited, eligible and willing to participate. Of those, 1738 lived in urban areas and 664 lived in rural areas.

### 2.2. Measures

Walking participation was self-reported from the previously validated Active Australia Survey [22]. Walking frequency was measured by asking, “*In the last week, how many times have you walked continuously, for at least 10 min, for recreation, exercise or to get to or from places?”* [23]. Perceived neighbourhood walkability was assessed using six items from the previously validated Neighbourhood Environment Walkability Survey-abbreviated version (NEWS-A) questionnaire, for which moderate test-retest reliability has been reported [24]. Two items were related to destinations, and four were related to routes and aesthetics. Each question was on a four-point Likert scale from one (‘strongly disagree’) to four (‘strongly agree’); higher scores indicated greater walkability. Three walkability scores were calculated: destination-related walkability (DW, mean of two items related to local destinations); route-related walkability (RW, mean of four items related to quality of walking routes and aesthetics); and overall walkability (OW, mean of all six items).

Demographic variables included age, sex, household income (<$40,000, $40,001–80,000, $80,001–100,000, >$100,000 or Refused/do not know), education (Secondary school or lower, Trade certificate/Apprenticeship/Certificate/Diploma or Bachelor degree or higher), work status (Employed (full or part time), Unemployed or Economically inactive), marital status (Married/Living with partner, Separated/Divorced/widowed or Never Married), body mass index (BMI) (calculated from self-reported height and weight and categorised according to World Health Organization definitions [25]), and self-reported health determined by the Short Form 1 (SF1) question (Excellent/Very Good, Good or Fair/Poor) [26]. The definition of urban and rural was determined by the data custodians, based on the distinction between the capital city region (urban) and rest of state (rural), which in turn is based on the Australian Bureau of Statistics remoteness classification of urban, rural and remote. Post codes in the capital city region were defined as urban and the remainder as rural [27].

### 2.3. Statistical Methods

In order to be representative of the South Australian population, data were weighted by age, sex, area (urban/rural) and probability of selection in the household using the 2011 Australian Bureau of Statistics census data and the number of listings in the telephone directory.

Urban and rural differences in walking participation (reporting one or more bouts of at least 10 min in the last week) were compared using chi-square tests. Walk frequency was non-normally distributed and was therefore described using median (interquartile range (IQR)): urban and rural differences were compared using Kruskal Wallis tests. 

The perception of neighbourhood walkability was calculated as the mean score for each domain of walkability and reported as mean (standard deviation (SD)), and urban and rural areas were compared using one-way Analysis of Variance (ANOVA).

The association between perceived walkability components (continuous) and walk frequency was tested using negative binomial regression models for urban and rural areas. Models were adjusted for age, employment status, BMI, marital status, SF1, education and household income. Further analyses stratified by sex were also conducted as physical activity predictors are typically different between sexes [7]. Data were analysed using IBM SPSS for Windows version 22.0 (IBM, Armonk, NY, USA).

## 3. Results

Table 1 describes the demographic profile of the sample. There were significant differences between urban and rural residents for age, education, household income, marital status, and BMI (*p* < 0.05).

There was a significant difference in walking participation between urban and rural respondents (see Table 2). A greater proportion of people in rural areas reported no walking participation (24.9%) compared to people in urban areas (16.8%). There was a significant difference in the walk frequency with urban participants walking more often than rural participants (*p* = 0.049). By sex, there remained a significant difference between urban and rural residents reporting no walking participation (men (*p* < 0.001) and women (*p* = 0.004)), but no differences in either men or women for walk frequency (*p* = 0.229 and *p* = 0.110, respectively) (Table 2).

For each perceived walkability item, there was a significant difference between urban and rural participants, with a higher proportion of those in urban areas strongly agreeing with all statements (i.e., higher walkability) compared to those in rural areas (*p* < 0.001) (Table 3). Similarly, urban residents reported greater mean perceived walkability scores in all three domains compared to rural residents (Table 3).

Table 4 provides the results for the association between perceived walkability and walking frequency. For urban participants, there was a significant association between perceived walkability and walk frequency for the OW score; each additional unit in OW was associated with an 11% higher walk frequency. For DW, for each unit increase of DW there was a 14% higher walk frequency in urban residents. There was no significant association of RW with walk frequency for urban residents, and no significant association of walking participation with any domain of perceived walkability for rural residents.

In urban men, but not women, there was a significant association of OW and DW with walking frequency but no association between RW and walking frequency. In rural men and women, there were no significant associations between any domain of perceived walkability and walking frequency (Table 4).

## 4. Discussion

This study aimed to determine differences in perceived walkability and the associations between walk frequency and walkability for persons living in urban and rural areas in South Australia. Urban residents walked more often and perceived their local area to be more walkable in terms of access to local destinations and quality of routes to destinations. Perceived walkability was associated with walking participation and walk frequency in urban areas, but not in rural areas. For specific perceived walkability components, destination-related walkability (access to utilitarian and recreational destinations) was associated with walking only among urban residents, but route-related walkability (well-connected streets, footpaths, and attractive neighbourhood) was not associated with walking participation in urban or rural areas.

A possible interpretation of the findings for destination-related walking in rural areas is that access to, or the distance from, local destinations has no bearing on walking among rural residents, potentially due to habitual use of cars to get to these destinations, or not having destinations within walkable distances. Perceived walkability was assessed for local areas around one’s residential address, but in rural areas, walking behaviour may have occurred away from the residential location.

As shown in Table 3, destination-related perceived walkability scores were significantly higher in urban areas. Previous studies have reported that walking for transport, which is reflective of walking to destinations, is lower for rural residents compared to urban residents [7] and that walking in rural areas is more likely leisure based than for transport [20]. In the current study, there was a difference between urban and rural residents in perceived walkability across all domains for men and women, with higher walkability scores in urban areas. Consequently, access to local destinations may not be a suitable measure for assessing rural walkability. Although a systematic review reported that availability of destinations was associated with walking for transport in 80% of identified studies [14], this may reflect the large proportion of urban studies in the review. It is possible that the domains for perceived walkability used in this study may not be appropriate in rural areas [20]. Further research should examine domain-specific walkability in rural areas.

There was no association between walking behaviour and route walkability among urban or rural residents, suggesting that having local places to walk to may have more influence on participation than attributes of the route. The relevance of route attributes to walking for transport has been shown in previous studies [28,29,30]. However, Sugiyama et al., 2012 demonstrated that route attributes were not consistently associated with walking for recreation, with only one third of studies reviewed reporting significant associations [14]. Thus, the non-significant relationships for route attributes used in this study may be due to walking participation combining both transport and recreation walking.

Although some studies have reported sex-related differences in walking behaviour [21], the results from this study did not find any significant associations between perceived walkability and walking behaviour in women in urban or rural locations. A study by Berry et al., in 2016 reported a similar non-significant association between recreational walking in women and place of residence, suggesting that what mattered for women’s recreational walking behaviour was more aligned with motivation to walk and not where they lived [7]. This result may also highlight the unsuitability of the perceived walkability domains in the survey to identify gender influences on walking behaviour. Perhaps there are other factors that are more important for gender walking behaviour that could be included in future studies, such as, social support (someone to walk with), weather [21], and perceived safety [31].

This study is reliant on self-reported walking, which can involve reporting bias and recall error [32]. The non-specific nature of the walking measure (grouping transport with recreational walking) is another limitation of the study, as walking for different purposes seems to be related differently to environmental attributes. Walkability was also assessed using self-reporting, although both perceptions of the environment and objectively-derived environmental attributes are known to be independently associated with walking [33]. Further research with purpose-specific walking and objective environmental measures is warranted. In addition, the proportion of households with no landlines (fixed telephones) is increasing, which has the potential to introduce bias into the sample [34]. To overcome bias, these data were weighted, which is a common statistical approach to overcome biases in survey data, so the results are reflective of the South Australian population.

## 5. Conclusions

This study demonstrated that the presence of local destinations seemed to facilitate more frequent walking among urban residents. However, walkability components found relevant to walking in previous studies (mostly conducted in urban areas) were not related to walking in rural areas. Rural residents’ walking may be influenced by unique contextual factors that warrant further research.

Since rural residents tend to be less active and have poorer health than urban residents [10], it is important to encourage walking in rural areas. More research is required to understand the environment attributes that are associated with walking behaviour in rural areas. What is clear from this study is that the perceived walkability measures focusing on destinations and routes were not salient for rural residents. The need to increase physical activity in urban and rural communities remains both a priority and a challenge, and is coupled with the need for better understanding as to how different environment attributes in urban and rural areas may facilitate walking participation. Without a better understanding of what sits behind the lower walking participation and walk frequency in rural areas, it is difficult to develop interventions or plan improved built environments to increase walking behaviours. Understanding environmental attributes that can encourage walking in rural settings would inform activity promotion initiatives in such areas.

## Figures and Tables

**Table 1:** Demographic profile of respondents.

	Total (N = 2402)	Urban (N = 1738)	Rural (N = 664)	*p*
Sex (male) ^a^	1164 (48.4)	834 (48.0)	330 (49.7)	0.24 ^a^
Age (years) (mean ± SD)	48.1 ± 18.4	47.8 ± 18.5	50.5 ± 17.9	0.001 ^b^
Employment (n (% employed))	1450 (60.0)	1042 (59.9)	409 (61.5)	0.243 ^a^
Education (n (% Secondary or lower))	962 (40.1)	646 (37.2)	316 (47.7)	<0.001 ^a^
Household income (n (% less than $40,000 pa))	439 (18.3)	303 (17.4)	136 (20.5)	0.003 ^a^
Marital status(n (%Never Married))	519 (21.7)	410 (23.7)	109 (16.5)	<0.001 ^a^
BMI (n (% overweight or obese))	1285 (57.2)	881 (54.1)	404 (65.4)	<0.001 ^a^
Self-reported health (n (% Fair/poor))	570 (23.7)	408 (23.5)	162 (24.4)	0.902 ^a^

^a^ Compared using chi square test; ^b^ Compared using Analysis of Variance.

**Table 2:** Number of times walked (for at least 10 min) per week and proportion of participants who report doing no walking by area of residence and sex.

	Urban		Rural		
	N	Value	N	Value	*p*
**Total sample**					
Median times walked (IQR)	1731	4.0 (5.0)	660	4.0 (6.0)	0.049 ^a^
No walking% (95% CI)	291	16.8 (15.1–18.7)	164	24.9 (21.7–28.3)	<0.001 ^b^
**Men**					
Median times walked (IQR)	829	4.0 (5.0)	327	4.0 (7.0)	0.229 ^a^
No walking% (95% CI)	150	18.1 (15.6–20.9)	90	27.6 (23.0–32.7)	<0.001 ^b^
**Women**					
Median times walked (IQR)	902	4.0 (5.0)	333	4.0 (6.0)	0.110 ^a^
No walking% (95% CI)	140	15.5 (13.3–18.1)	74	22.2 (18.1–27.0)	0.004 ^b^

^a^ Compared using Kruskal Wallis Test; ^b^ Compared using chi square test.

**Table 3:** Walkability scores by area of residence.

	Urban		Rural		
	N	Mean (SD)	N	Mean (SD)	*p*
**Destination-related Items ^a^**					
There are many shops or other places to buy things I need within easy walking distance of my home	1733	2.83 (1.11)	663	2.21 (1.23)	<0.001 ^b^
There is a park or nature reserve in my local area that is easy to get to	1732	3.62 (0.79)	660	3.02 (1.21)	<0.001 ^b^
**Route-related Items ^a^**					
There are many alternative routes for getting from place to place when walking in my local area	1721	3.08 (0.98)	661	2.61 (1.21)	<0.001 ^b^
There are footpaths on most of the streets in my local area	1736	3.42 (0.94)	664	2.56 (1.29)	<0.001 ^b^
There are bicycle or walking tracks in or near my local area that are easy to get to	1684	3.17 (1.09)	653	2.66 (1.27)	<0.001 ^b^
My local neighbourhood is attractive (buildings, trees, household gardens)	1736	3.48 (0.71)	663	3.27 (0.92)	<0.001 ^b^
**Mean Walkability Scores**					
**Total**					
Overall Walkability	1666	3.27 (0.59)	647	2.72 (0.59)	<0.001 ^c^
Destination Walkability	1728	3.23 (0.77)	660	2.61 (1.00)	<0.001 ^c^
Route Walkability	1670	3.29 (0.61)	650	2.77 (0.82)	<0.001 ^c^

^a^ Item responses on 4-point Likert scale, 1 = strongly disagree, 4 = strongly agree; ^b^ Compared using chi square test; ^c^ Compared using analysis of variance.

**Table 4:** Association between perception of walkability and walking frequency for urban and rural residents, for total sample and stratified by sex.

	Urban			Rural		
	n	IRR (95% CI)	*p*	n	IRR (95% CI)	*p*
**Total**						
Overall Walkability	1576	1.11 (1.01–1.22)	0.030	534	1.00 (0.89–1.11)	0.923
Destination Walkability	1692	1.14 (1.06–1.22)	<0.001	544	0.97 (0.89–1.07)	0.541
Route Walkability	1581	1.05 (0.96–1.15)	0.277	536	1.11 (0.91–1.14)	0.798
**Men**						
Overall Walkability	811	1.16 (1.02–1.33)	0.024	294	0.98 (0.84–1.15)	0.811
Destination Walkability	827	1.24 (1.12–1.37)	<0.001	298	0.97 (0.85–1.10)	0.965
Route Walkability	813	1.06 (0.93–1.20)	0.377	295	1.00 (0.85–1.17)	0.997
**Women**						
Overall Walkability	765	1.04 (0.91–1.19)	0.599	240	1.09 (0.92–1.30)	0.335
Destination Walkability	802	1.03 (0.93–1.13)	0.569	246	1.05 (0.91–1.22)	0.480
Route Walkability	768	1.04 (0.91–1.19)	0.597	241	1.10 (0.93–1.32)	0.268

Analysed using negative binomial regression, adjusted for age, work status, BMI, self-reported health, marital status, education and income. IRR: incidence rate ratio.

## References

[1] Bauman A.E. (2004). Updating the evidence that physical activity is good for health: An epidemiological review 2000–2003. J. Sci. Med. Sport.

[2] Brown W.J. (2004). Physical activity and health: Updating the evidence 2000–2003. J. Sci. Med. Sport.

[3] Hamer M., Chida Y. (2008). Walking and primary prevention: A meta-analysis of prospective cohort studies. Br. J. Sport. Med..

[4] Lee Y.-S. (2005). Gender differences in physical activity and walking among older adults. J. Women Aging.

[5] Morris J.N., Hardman A.E. (1997). Walking to health. Sport. Med..

[6] Lee I.-M., Buchner D.M. (2008). The importance of walking to public health. Med. Sci. Sport. Exerc..

[7] Berry N., Smith M., Ullah S., Dollman J. (2016). Walking for recreation and transport by geographic remoteness in south australian adults. Aust. J. Rural Health.

[8] Van Dyck D., Cardon G., Deforche B., De Bourdeaudhuij I. (2011). Urban–rural differences in physical activity in belgian adults and the importance of psychosocial factors. J. Urban Health.

[9] Australian Bureau of Statistics (2013). Australian Health Survey: Physical Activity, 2011-12. Catalogue Number: 4364.0.55.004.

[10] Australian Institute of Health and Welfare (AIHW) (2014). Australia’s Health 2014. Australia’s Health Series No. 14. Cat. No. Aus 178.

[11] Owen N., Cerin E., Leslie E., Coffee N., Frank L.D., Bauman A.E., Hugo G., Saelens B.E., Sallis J.F. (2007). Neighborhood walkability and the walking behavior of australian adults. Am. J. Prev. Med..

[12] Van Dyck D., Cardon G., Deforche B., Sallis J.F., Owen N., De Bourdeaudhuij I. (2010). Neighborhood ses and walkability are related to physical activity behavior in Belgian adults. Prev. Med..

[13] Hajna S., Ross N.A., Brazeau A.-S., Bélisle P., Joseph L., Dasgupta K. (2015). Associations between neighbourhood walkability and daily steps in adults: A systematic review and meta-analysis. BMC Public Health.

[14] Sugiyama T., Neuhaus M., Cole R., Giles-Corti B., Owen N. (2012). Destination and route attributes associated with adults’ walking: A review. Med. Sci. Sport. Exerc..

[15] Frost S.S., Goins R.T., Hunter R.H., Hooker S.P., Bryant L.L., Kruger J., Pluto D. (2010). Effects of the built environment on physical activity of adults living in rural settings. Am. J. Health Promotion.

[16] Saelens B.E., Handy S.L. (2008). Built environment correlates of walking: A review. Med. Sci. Sport. Exerc..

[17] Kegler M.C., Swan D.W., Alcantara I., Feldman L., Glanz K. (2014). The influence of rural home and neighborhood environments on healthy eating, physical activity, and weight. Prev. Sci..

[18] Sugiyama T., Cerin E., Owen N., Oyeyemi A.L., Conway T.L., Van Dyck D., Schipperijn J., Macfarlane D.J., Salvo D., Reis R.S. (2014). Perceived neighbourhood environmental attributes associated with adults׳ recreational walking: Ipen adult study in 12 countries. Health Place.

[19] Carr L.J., Dunsiger S.I., Marcus B.H. (2010). Validation of walk score for estimating access to walkable amenities. Br. J. Sport. Med..

[20] Kegler M.C., Alcantara I., Haardörfer R., Gemma A., Gemma D., Gazmararian J. (2015). Rural neighborhood walkability: Implications for assessment. J. Phys. Activ. Health.

[21] Humpel N., Owen N., Iverson D., Leslie E., Bauman A. (2004). Perceived environment attributes, residential location, and walking for particular purposes. Am. J. Prev. Med..

[22] AIHW (2003). The Active Australia Survey: A Guide and Manual for Implementation, Analysis and Reporting.

[23] Rzewnicki R., Auweele Y.V., De Bourdeaudhuij I. (2003). Addressing overreporting on the international physical activity questionnaire (IPAQ) telephone survey with a population sample. Public Health Nutr..

[24] Cerin E., Saelens B.E., Sallis J.F., Frank L.D. (2006). Neighborhood environment walkability scale: Validity and development of a short form. Med. Sci. Sport. Exerc..

[25] World Health Organisation (2012). Obesity and Overweight.

[26] Gill T.K., Broderick D., Avery J.C., Dal Grande E., Taylor A.W. (2009). Self reported overall health status: Implications for intervention strategies. Australas. Med. J..

[27] Dal Grande E., Taylor A., Wilson D. (2000). South Australian Health & Wellbeing Survey.

[28] Forsyth A., Hearst M., Oakes J.M., Schmitz K.H. (2008). Design and destinations: Factors influencing walking and total physical activity. Urban Studies.

[29] Knuiman M.W., Christian H.E., Divitini M.L., Foster S.A., Bull F.C., Badland H.M., Giles-Corti B. (2014). A longitudinal analysis of the influence of the neighborhood built environment on walking for transportation: The reside study. Am. J. Epidemiol..

[30] Koohsari M.J., Sugiyama T., Lamb K.E., Villanueva K., Owen N. (2014). Street connectivity and walking for transport: Role of neighborhood destinations. Prev. Med..

[31] Ball K., Timperio A., Salmon J., Giles-Corti B., Roberts R., Crawford D. (2007). Personal, social and environmental determinants of educational inequalities in walking: A multilevel study. J Epidemiol. Community Health.

[32] Sallis J.F., Saelens B.E. (2000). Assessment of physical activity by self-report: Status, limitations, and future directions. Res. Q. Exerc. Sport.

[33] McGinn A.P., Evenson K.R., Herring A.H., Huston S.L., Rodriguez D.A. (2007). Exploring associations between physical activity and perceived and objective measures of the built environment. J. Urban Health.

[34] Dal Grande E., Taylor A. (2010). Sampling and coverage issues of telephone surveys used for collecting health information in australia: Results from a face-to-face survey from 1999 to 2008. BMC Med. Res. Methodol..

